# Vocational rehabilitation via social firms: a qualitative investigation of the views and experiences of employees with mental health problems, social firm managers and clinicians

**DOI:** 10.1186/s12888-021-03577-5

**Published:** 2021-11-12

**Authors:** Nicola Morant, Alyssa Milton, Eleanor Gilbert, Sonia Johnson, Nicholas Parsons, Swaran Singh, Steven Marwaha

**Affiliations:** 1 Division of Psychiatry, University College London, Maple House, 149 Tottenham Court Road, W1T 7NF London, UK; 2 Faculty of Medicine and Health, University of Sydney, Sydney, Australia; 3 Division of Mental Health and Wellbeing, Warwick Medical School, University of Warwick, Coventry, UK; 4 Camden and Islington NHS Foundation Trust, London, UK; 5 Statistics and Epidemiology Unit, Warwick Medical School, University of Warwick, Coventry, UK; 6 Institute for Mental Health, School of Psychology, University of Birmingham, Birmingham, UK; 7 Birmingham and Solihull Mental Health Trust, Birmingham, UK

**Keywords:** Vocational rehabilitation, Supported employment, Social firms, Social enterprise, Mental illness, Qualitative research, UK

## Abstract

**Background:**

Employment within social firms in the UK is under-developed and under-researched, but a potentially beneficial route to vocational rehabilitation for people with mental health problems. This study explores the views and experiences of employees with mental ill-health, managers of social firms and mental health clinicians, in order to understand the potential value of social firms for the vocational rehabilitation, employment and well-being of people with mental health problems.

**Methods:**

Semi-structured interviews were conducted with 23 employees with mental health problems in 11 social firms in England. A focus group and individual interviews were conducted with 12 managers of social firms. Two focus groups were held with 16 mental health clinicians. Data were analysed using thematic analysis.

**Results:**

Most employees expressed very positive views about working in a social firm. In responses from both employees and social firm managers, an overarching theme regarding the supportive ethos of social firms encompassed several related features: openness about mental health issues; peer, team and management support; flexibility; and support to progress and develop skills over time. Managers identified benefits of employing people with mental health problems who were sufficiently recovered. Knowledge of social firms within clinician focus groups was very limited, although clinicians thought they could be a welcome additional vocational resource.

**Conclusions:**

High levels of job satisfaction among social firm employees may be explained by the supportive ethos of these working environments. Social firms have potential to be a helpful addition to the range of vocational pathways available for people with mental ill-health. Further mixed methods investigations of experiences and outcomes in order to understand who engages with and benefits from this form of vocational rehabilitation would be valuable in informing decisions about scaling up the model.

**Supplementary Information:**

The online version contains supplementary material available at 10.1186/s12888-021-03577-5.

## Background

Employment is widely recognised as an important element in the multi-faceted process of recovery from mental ill-health [1, 2]. However, employment rates for people with serious mental illnesses are low across Europe [3–5]. This may be attributable to numerous barriers including lack of choice, opportunity and work-place support, stigma, and disincentives to employment in the welfare system [6–8].

The most widely advocated type of supported employment for those recovering from mental health problems is individual placement and support (IPS) in which support is provided to search for competitive open market employment, and subsequently to employees and employers in maintaining employment once a job has been obtained [9]. Meta-analyses and systematic review evidence has shown that, internationally, IPS has beneficial effects on employment outcomes compared with other vocational services (e.g. [9–13]). In the United Kingdom (UK), IPS is recommended in national clinical guidelines for schizophrenia, psychosis and complex psychosis [14, 15]. Although IPS is feasible in the UK [16] a problem common to the UK and several other European countries is implementation. Problems including fidelity, uptake, engagement and sustainability of employment have been identified [17–20], and IPS has had less success at improving outcomes compared to those reported in the USA [21, 22]. This difference has been attributed to the specifics of local economic, health and welfare contexts and the scale of IPS implementation [22–24].

A complementary vocational support model to IPS may be provided by social firms. Social firms are well-established in North America, Australia and mainland Europe. They are types of social enterprise with a defining criteria of supporting and empowering disadvantaged people by drawing at least 25% of their workforce from groups facing barriers to mainstream employment, including people with mental health problems [25]. Sometimes known as affirmative or social businesses in the USA, or as integrated cooperatives or work integration social enterprises in Europe [25–29], they are competitive businesses, with disadvantaged employees paid at market rates, and integrated with other workers. Pathways into social firm employment for people with mental health problems vary, and may be via an employment agency, mental health services or open market adverts. Similarly, eligibility criteria vary, although identification of mental health problems by primary or secondary services is typical. Managers are aware that employees have previously been, or are currently impacted by mental ill-health.

There is international evidence that social firms can provide sustainable employment and promote social inclusion whilst operating as prosperous businesses [28, 30], although many also receive government subsidies that assist their financial viability whilst also supporting disadvantaged groups [31]. They typically offer more workplace accommodations and forms of support to employees than mainstream work environments [30, 32, 33]. High job satisfaction among employees with mental health problems has been reported in Italian social firms, linked to workplace accommodations and support from co-workers [34]. A handful of small qualitative studies in social firms (or similar organisations) have identified positive impacts for employees of a supportive culture, workplace relationships, a sense of personal competence, and a ‘normalising life-world’ [35–39]. Only one of these was in the UK [37], many focus on a single social firm, and none include the perspectives of other relevant stakeholders.

While social firms seem promising as a means of extending the range of vocational support, they are under-developed in the UK, particularly compared to other European countries, and under-researched [40]. In a previous survey, we found 33 UK social firms that empoyed people with mental health problems, 50% employing people with schizophrenia or bipolar disorder [31]. These were small businesses employing 15 people on average, and were predominantly in the manufacturing, service, recycling and catering sectors. We also found strikingly high levels of job satisfaction among employees with mental health problems [41], above those reported in the general population [42]. This paper reports on an in-depth, qualitative study that aims to explore the reasons for these high levels of reported job satisfaction, and the utility of social firms in helping people with mental health problems return to and sustain employment in the UK context. Our study improves on previous work in this area by collecting data in multiple sites, and from multiple stakeholder perspectives. It explores and compares the views and experiences of social firm employees with mental health problems, social firm managers and mental health clinicians.

## Methods

Methods are reported in line with COREQ guidelines [43] and a completed 32 item checklist containing more details is provided in Appendix 2 (supplementary information).

### Setting

This qualitative study was part of a broader research project investigating the value of social firms in the UK in the vocational recovery of people with mental health problems [31, 41].

### Participants and data collection

Recruitment of social firm employees and managers was via social firms in England and Wales identified in our earlier survey [31]. The study was advertised through social firm communications from managers at each site, and interested participants were invited to contact study researchers or their manager. Semi-structured interviews and focus groups were conducted with three groups:

#### Social firm employees with mental health problems

A semi-structured interview schedule was designed to explore employees’ experiences of working in a social firm. Questions covered perceived benefits and problems, recruitment and support mechanisms, impact on other areas of life, comparisons with previous work experiences, and aspirations for the future (Appendix 1). In order to enhance relevance, rapport and sensitivity, trained service user researchers contributed to interview design and conducted interviews. Eight service user researchers with personal experience of mental ill-health received training from members of the study team on qualitative interviewing techniques. Four pilot interviews were conducted to ensure interviewer competency and appropriateness of the schedule, and service user researchers received ongoing support and supervision. Interviews were conducted in private rooms at employees’ workplaces. They lasted approximately one hour and were audio-recorded and transcribed verbatim. Participants received monetary compensation for their time (£20). Eligibility criteria were employment in a social firm in England or Wales, and self-identified experience of mental ill-health.

#### Managers

Managers of social firms in England and Wales where at least one person with mental health problems was employed were invited to participate in a focus group facilitated by two study researchers (AM and EG). Topics included the benefits and problems of social firm employment for people with mental health problems and for the social firm, and links with mental health organisations (Appendix 1). Individual telephone interviews covering the same topics were conducted by the same researchers with managers who were unable to attend the focus group. Interviews were recorded and transcribed. Detailed notes of the focus group discussion were taken and their accuracy was checked with participants.

#### Mental health clinicians

Clinicians were recruited from community mental health teams in the Midlands of England and London to participate in two focus groups conducted by AM and EG. Recruitment was via mental health team managers who were asked to approach clinicians from a range of professional backgrounds. Topics included clinicians’ awareness of local social firms and experiences of supporting service users to work there, how social firms compare to other vocational models, and the suitability of social firms for their service users (Appendix 1). Discussions were audio-recorded and transcribed.

Sample sizes for the three groups of participants were planned based on published guidance [44]. As we wanted to prioritise the experiences of social firm employees and to include the perspectives of people working in a range of employment sectors, and with variable lengths of employment and forms of mental ill-health, we planned to interview up to 30 social firm employees, depending on data quality and saturation. For social firm managers and clinicians we used guidance of around 12 as a minimum sample size [44]. Confidentiality and anonymity were discussed before data collection and informed consent was obtained from all participants.

### Data analysis

Data were analysed using thematic analysis [45] within Nvivo10 software. Analysis combined inductive and deductive approaches, allowing exploration of both initial research questions and themes from participants’ own experiences. Following the broad principles and stages of thematic analysis, data codes, themes, and subthemes were iteratively developed and refined throughout the analytic process. Data from stakeholder sub-groups (employees, managers and clinicians) were analysed separately, then brought together and compared in later stages of analysis. In order to enhance validity, a collaborative approach was adopted. NM, AM and EG coded the data, and a small team of other researchers including a service user researcher contributed to reading transcripts and coding frame development, and held regular discussions about emerging themes.

## Results

### Participants

Data were collected from employees and managers of 14 social firms (3 employees only; 3 manager only; 8 managers and employees). Interviews were conducted with 23 social firm employees across 11 social firms. Data collection ceased when the research team considered that data saturation had been reached. The modal number of people interviewed per firm was 2 and the maximum was 4. Social firms were in a range of sectors: recycling (2), training (2), and one each of: printing, gardening, health foods, market research, travel agent, framing and textiles. Firms were generally small, employing on average 7 people in total and 3 with mental health problems. They were all in England, and distributed as follows: South east *n* = 5; south / south west *n* = 1; midlands *n* = 4; north *n* = 1. Participants’ demographic and mental health characteristics are shown in Table 1. Employment circumstances are shown in Table 2.

**Table 1:** Demographic and mental health characteristics of social firm employees

	*N* = 23
Gender	
*Male*	12
*Female*	11
Age (*n* = 20): mean	48.6 (range: 32–64)
Marital status	
*Unmarried*	9
*Married*	9
*Cohabiting*	1
*Separated/divorced*	4
Diagnosis (n = 20):	
*Depression/anxiety*	17
*Schizophrenia/psychosis*	3
Previous hospital admission for mental health treatment (*n* = 20)	
*Yes*	10
*No*	10
Currently using mental health services (*n* = 20)	
*Yes*	14
*No*	6

**Table 2:** Current employment features of social firm employees

	*N* = 23
Hours of work (*n* = 21):	
*Full time*	6
*Part time*	15
*Mean hours worked*	24.2
Contract type (*n* = 21):	
*Permanent*	16
*Fixed term*	5
Length of time at social firm (mean in months)	56.9 (Range 4–192)
Previous voluntary work at social firm:	
*Yes*	15
*No*	8
Been promoted at social firm? (*n* = 21)	
*Yes*	14
*No*	7
Occupational category*	
*Professional*	0
*Associate professional/technical*	3
*Skilled trade*	5
*Administrative/secretarial*	2
*Elementary*	6
*Manager/director*	3
*Sales/customer services*	4

* Based on Office for National Statistics: Standard occupational classifications 2000

Twelve social firm managers participated (7 in the focus group, 5 in individual interviews). Sixteen mental health clinicians from 5 community mental health teams (2 in the Midlands of England, 3 in London) took part in focus groups. They worked in early intervention, assertive outreach, and recovery and rehabilitation services. The sample comprised 4 community psychiatric nurses (CPNs), 4 consultant psychiatrists/registrars, 4 occupational therapists (OTs), 3 team managers and 1 student nurse.

### Qualitative themes

Social firm employees and managers provided ‘inside’ perspectives on working in social firms and there were many thematic similarities in their data. Accordingly, findings from employees and managers are presented together, followed by analysis of clinicians’ ‘outside’ perspective.

### The supportive ethos of social firms

Managers generally saw social firms as suitable for people with a range of mental health problems, as long as they were sufficiently recovered from an acute phase of illness. Perceived benefits of employing people with mental health problems included their commitment, skills and experience. Managers also discussed tensions between providing a supportive environment and running a viable business, and the additional responsibility of monitoring and managing mental health issues in the workplace.

*“Once you get someone in an environment where they feel supported, trusted, comfortable, etc., they’ve got all that energy, all that drive [ … ] What you generally get is a very enthusiastic, motivated employee.”* [M3]^1^

*“You’ve still got all the same things that you have with a traditional profit making business, but you get this added thing occasionally where somebody’s health takes a dip. And then suddenly you have to focus on that person and try and work out what’s going on, when you’ve still got all the pressures of meeting deadlines and trying to keep the turnover up, so it can be challenging as a manager.”* [M2]

The majority of employees were very positive about working in a social firm. This was related to an overarching theme of their supportive ethos that united much of what both employees and managers said, and was often contrasted with experiences of mainstream employment

*“It’s nice that the ethos is … you’re being encouraged to be independent, the help is there if you need it but, if we give you a little push, you can do this ... which you do need, if you’ve got mental health problems, you do need someone to give you a bit of a push to say, come on, you can do this.”* [E17]

**Fig. 1:**
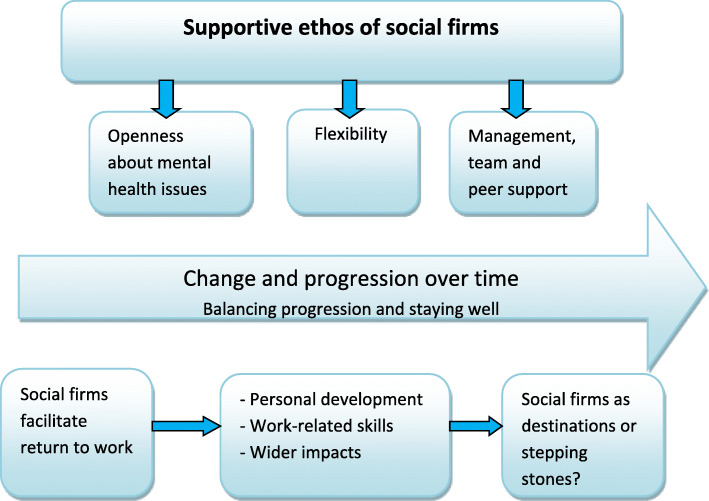
Social firms as a supportive work environment

This overarching theme encompassed several specific and related issues that are described in more detail below: openness about mental health issues, support from managers and colleagues, flexibility, and support to progress and develop skills over time (Fig. 1).

#### Openness about mental health issues

An important feature of the supportive ethos of social firms was openness about mental health issues that encouraged initial disclosure and subsequent acceptance. Employees reported being encouraged to seek support from managers, most of whom had training in mental health awareness, and/or a history of mental ill-health themselves. Several social firms had links with local mental health services, liaising with services to support employees if their mental health became a cause for concern. Most employees felt they could be open about their mental ill-health without fear of negative consequences and were aware of colleagues’ mental health issues. Employees felt valued rather than stigmatised because of their mental health problems. This was often contrasted to previous workplace experiences that were seen as contributing to mental ill-health.

*“At previous jobs my anxiety was made worse by the fact that I knew that no-one understood. … No-one had any empathy over why I might be being how I was being. And that is completely alleviated working at [social firm], because I know that if I’m having an off-day, no one will judge me on it, or question it.” [E7]*

#### Flexibility

Another manifestation of the supportive ethos of social firms was flexibility, both in the long-term (e.g. changes to contracted hours) and in day-to-day organisation. Employees described working the number of hours that suited them, working patterns to help them manage mental health symptoms, and flexibility to change tasks, take a break, change working days or take time off for appointments. Several managers described how flexibility and support were built into the structure of the social firm.

*“At each stage I’m always, even now, asked whether or not I can cope with it, if I want to do it. … Very often I’ll say, yes I’ll do it. And then I’ll find I can’t, they’re very good in saying, well look it doesn’t matter, maybe we’ll come back to it or maybe someone else will do it. So they’re very flexible and understanding with regards to gradually increasing what I’m doing.” [E13]*

*“ … .because of the way we’re structured, it enables people to have a much more flexible working practice. Because when [firm name] was started, it was started by people with a mental health background, both as service users and people who had worked professionally in mental health.” [M4]*

A small number of employees described flexibility less positively, as allowing them to avoid responsibility. Two respondents felt they would benefit from managers being more directive, setting limits or encouraging them to continue with work despite mental health issues.

#### Management, peer and team support

Support and understanding from managers was discussed by all employees as an important aspect of their work experience. For some, this made the difference between being able to work or not, or enabled them to have shorter periods of time off work if they became unwell.

*“Last year I had a few weeks off because I took an overdose in April, that’s why I ended up in hospital. And our MD was really understanding about that. [ … ] I didn’t think being at home alone helped me in my recovery from that, but I was struggling with my anxiety with leaving the house. So our MD picked me up every morning.” [E7]*

Again, this was compared positively with previous work experiences in which lack of management support was frequently perceived as contributing to mental ill-health. Employees also described relying on colleagues if they had problems. This was often discussed in relation to shared experiences and understandings of mental ill-health or other difficulties, and was associated with a strong sense of team identity and belonging that was an important part of employees’ job satisfaction. There were some suggestions that the small size of social firms facilitated this strong team identity.

*“I’ve never worked for a place like it. We all support each other. We all look out for each other. If you’ve got a problem I know I can say, oh, I’m not very good today, or can I swap this, or maybe I need to do that, and you know that network is there. There’s support there so you don’t have to pretend.” [E4]*

### Dynamic issues: change and progression over time

An important part of the supportive ethos of social firms described by both employees and managers was the encouragement to develop and progress over time in both work-related and personal domains. There was a perceived need to balance this with strategies to preserve well-being or prevent the re-emergence of mental ill-health. Employees described encouragement to expand skills or take on additional responsibilities at a pace that suited them, but did not feel under pressure to do this. For some, this was seen as part of a wider and long-term recovery journey, and linked to broader impacts of work in a person’s life.

*“It’s a fine balance with giving you more responsibility to help you in your recovery, without giving you so much responsibility that they wreck your recovery and you go back to square one, because that’s what it’s about, it’s about the slow steady process of recovery.” [E16]*

#### Social firms facilitate return to work

Recruitment experiences were generally described positively and often as less formal than elsewhere, with disclosure of mental health problems being less problematic than in other contexts. Most respondents felt allowances for their mental health had been made, for example, having a ‘taster’ work session rather than a formal interview, or starting work in a voluntary capacity before progressing to paid work. Managers saw building self-confidence as central to recruiting people with mental health problems. Some employees felt the social firm had enabled them to return to work at an earlier stage.

*“Moving straight away into proper paid work would have been a little bit too stressful for me in the state I was in then. I was just getting used to being able to work, being able to meet people again, being out in the world, as it were, and suddenly being thrown into paid employment - I couldn’t have coped with it. … So they eased me into it gently. So it wasn’t quite so frightening when they said well, we do have this paid post.” [E16]*

*“I think it’s a starting place for people that have been out of work for some time. It’s a gentle way back into the workplace … .for example if somebody can start off as a volunteer, or on one or two days a week and then build up. I suppose that is all part of the flexibility, I think it’s the way that social firms like ours enable, show people a way back into employment that is a different model. I think that a lot of the benefits are around building up confidence and starting to feel better, and starting to feel I can do this again.” [M4]*

#### Wider impacts

Employees attributed a number of positive effects on their broader lives to working in a social firm, particularly in mental health, relationships and social networks. These included developing social skills, increasing confidence and self-esteem, feeling “normal”, and pride associated with earning money and not relying on welfare benefits.

*“If you feel low and you dislike yourself and you’ve suffered badly from anxiety, you need to have something where you can just see that you are achieving and see that you are okay. That in turn means you’re able to form better, more relaxed relationships with other people that you meet. I think it’s very, very important for people in recovery to have that.”[E12]*

#### Social firms as stepping stones or destinations?

There was some variability among the views of employees and managers over whether social firms are stepping stones back to more mainstream employment, or permanent employment destinations in themselves. The majority of employees wished to continue working at their social firm, and viewed their jobs as permanent. A smaller number saw the social firm as a stepping stone to other employment, with a few expressing less satisfaction about their current working roles.

*“My ambition hopefully is to stay with the [social firm] because it’s secure, the staff are good, there’s good camaraderie most of the time between us. Both [manager] and [deputy] are very understanding and supportive, and I am also very loyal to his ethos of [the social firm]. I mean, there are some people who have moved on, not many.”[E8]*

*“I’m not looking for anything else, but what this job has helped me to do is to put myself in a position where I feel like I can look for something else, that I could do something else.” [E14]*

### Clinicians’ views

Knowledge and experience of social firms within the clinician focus groups was very limited. No participants knew previous or current service users who had worked in a social firm. Clinicians were generally positive about the social firm model of employment and said they would welcome their availability as an additional vocational resource. They could see value in the provision of embedded, ongoing support and links with the mental health sector. Some saw social firms as more likely to allow use of previous work experience and skills than other vocational schemes.

*“I like the sound of this because it sounds like it’s not just about planting seeds and peeling potatoes and that, because that’s what we’re dragging some people down doing. Because that’s all we’ve got… that’s the only resource we’ve got.” [CFG1]*

A few clinicians thought that stigma associated with the agenda to support socially disadvantaged groups might be off-putting to some (and were aware of similar service user concerns about IPS). Although clinicians saw value in vocational schemes that enabled people to be “work ready” (sufficiently recovered from mental health problems, motivated and able to manage daily life), currently available schemes (vocational training, work experience placements and IPS) were seen as rarely resulting in sustained paid employment:

*“ … .it’s perhaps, for a while, been positive for the service user involved, but there doesn’t seem to be an end point at which they can suddenly find a job or even a few hours work. And there’s been a dwindling pool of these type of resources anyway, and so I would say that probably the clients that we’ve had that have actually found employment as a consequence of any placement they’ve been on is really, they’re few and far between.” [CFG2]*

Some clinicians had experienced problems in continuing to monitor and maintain contact with service users once they had taken up employment. They felt that the opportunity to build links with a local social firm may help alleviate this problem.

*“It’s always a bit of a worry because we’re not too sure what’s going on there, and there’s a fine line between being nosy and being concerned. But I think if the person’s still under secondary health services, then we do want to try and keep an eye on them.” [CFG3]*

## Discussion

### Principal findings

People with mental health problems employed in UK social firms provided generally very positive views of their experiences in these contexts. This is consistent with findings of our larger quantitative study in which high levels of job satisfaction were reported [41]. Qualitative themes suggest this may be accounted for by the generally supportive ethos of social firms, specifically atmospheres of openness and acceptance about mental health issues, flexibility, and strong peer and management support. There are similarities with the findings of other research on social firms, including both survey work [30, 32, 33] and smaller qualitative studies [35–38, 46]. In particularly, the importance of social support, workplace accommodations, and an accepting working environment as defining features of social firms is highlighted internationally in this body of work.

In order to understand these findings in broader context, particularly in relation to IPS, the most common form of vocational rehabilitation for mental health service users in the UK and other high income countries, it is important to consider the clinical and work profiles of those in our sample. Nearly half of respondents (44%) reported a previous psychiatric hospital admission, 61% were currently using mental health services, and reported diagnoses were predominantly depression or anxiety, with smaller numbers reporting schizophrenia / psychosis. This suggests a range of moderate to severe previous or current mental health problems, and a clinical population that overlaps with those who typically receive IPS.

The majority of employees in this study worked part-time, had worked voluntarily before becoming paid employees, and had been promoted within the social firm. Employees told us they valued the progressive model offered by social firms, in which there was initially less emphasis on being ‘work ready’, and opportunities to gradually build up hours and responsibilities. Managers saw a certain level of recovery and stability as necessary before employment could be offered. The flexibility and support offered within social firms meant that when employees had suffered mental health wobbles or relapses, they found it easier to remain in work, or described earlier and easier returns to work than in other work contexts. The average length of employment at time of interview was over 4.5 years, and most held permanent work contracts and wanted to remain with the social firm rather than progress to other employment. Taken together, these features suggest a long-term and dynamic model of vocational support, compatible with the recovery concept involving both progresses and set-backs [1]. While social firms are a stepping stone to other employment for some, for many they provide more long-term employment. This contrasts with IPS in which average reported job tenure is much shorter [47], and support is targeted more towards initial (re-)entry into the workplace.

Managers described how the supportive social firm ethos was incorporated into organisational policies and recruitment procedures. Their view of vocational support was more resource-oriented than deficit-oriented: they saw employees with mental health problems as committed, loyal and often bringing relevant existing skills. However, managers acknowledged tensions between providing a supportive environment and running a profitable business, and additional responsibilities of monitoring mental health and liaising with mental health services. The low awareness of social firms among clinicians was striking. Within a context of expressed dissatisfaction with the suitability and limited availability of existing vocational resources, clinicians were generally positive about the social firm model, and in particular their working relationships with mental health services.

### Strengths and limitations

This study collected data from employees and managers in 14 of the 33 UK social firms employing people with mental health problems we previously identified [31], and has triangulated the views of three key stakeholder groups – employees with mental health problems, managers and mental health clinicians. Being both multi-site and multi-perspective, it is the most comprehensive qualitative study to date to investigate views and experiences of social firms as a vocational resource for people with mental health problems. People with lived experience of mental health problems collaborated and brought this perspective into the research, consulting on interview topics, conducting interviews and contributing to data analysis. Service user involvement has been called for in research in this domain [46], and is recognised as an important element of mental health research that can enhance data quality and the validity of findings [48, 49].

Limitations include only accessing the views of employees of social firms that had already agreed to participate in our research, and the self-selecting sample. We recognise therefore that the overwhelmingly positive views of social firm employment we heard in this research may be, in part, a reflection on recruitment processes. Similarly, those with less positive experiences may not have wished to participate in this research, or may no longer work at these social firm. Our findings may therefore be overly shaped by longer-term employees who have settled well into the social firm employment. Although the study sample is comparable to that of our larger quantitative study of UK social firms on demographic and clinical characteristics [41], it includes fewer people with schizophrenia or psychosis diagnoses (13% compared to 29%). We did not collect data on the demographic or professional characteristics of social firm managers so are not able to comment on whether the views we obtained from this group are typical of other social firm managers.

### Clinical, research and policy implications

People with mental health problems may struggle to return to mainstream employment after long periods out of work [50], and face dilemmas about disclosing mental health problems [8, 51]. We found low awareness of social firms among mental health clinicians seeking to support service users in vocational rehabilitation. Enhancing local partnerships in areas where social firms exist, and increasing awareness of alternatives to IPS among mental healthcare providers is suggested. While IPS has been the principal vocational rehabilitation model internationally, it implementation in the UK and elsewhere has been problematic, particularly in relation to employment sustainability [17]. Social firms provide a different vocational rehabilitation model in which longer job tenure appears to be related to a workplace culture where accommodations and support are central [32, 33]. They can provide longer-term employment within a dynamic model of vocational rehabilitation that is valued by many. Paradoxically however, if employees are reluctant to move on, the number of people who are able to benefit is limited. Additionally, if they are only destinations rather than stepping stones to other employment, there may be a risk of associated stigma, although evidence in the Italian context suggests this is not the case [52]. There are currently very few social firm in the UK, and they are generally small (a feature that may enable the supportive connections with peers and managers that employees value). They may therefore currently be successful in providing supportive and sustainable vocational rehabilitation for a few, but without substantial expansion of the social firm sector, numbers will remain small. The scalability of this form of vocational support should be investigated further. There is potential value in expanding the sector to match the scale of social firms seen in Europe, North America and Australia.

Managers in our study identified some tensions between providing a supportive environment and the financial viability of social firms. Financial support (from state, charity or mainstream business sources) may be needed to sustain social firms through periods of economic downturn, and to increase their numbers. The promotion of social enterprises (of which social firms are a form) can result in reduced public expenditure and increased revenues generated through tax, when compared with other interventions that seek to address social needs [53], and there have been calls for greater investment in social enterprises to support public mental health [54]. A stronger evidence-base for social firms is needed to enhance the case for supportive funding and investment. In particular, larger scale quantitative studies of long-term mental health and social functioning outcomes, and realist reviews to explore what works for whom and in which contexts are recommended. The viability of social firm employment for those who have previously struggled to sustain employment, and people with more enduring mental health problems who are particularly vulnerable to long-term unemployment should also be explored further.

## Conclusions

A variety of vocational rehabilitation pathways may be needed to meet the range of needs and provide choice for people with mental health problems returning to work [8, 20]. Although more research is needed to assess whether the social firms model has a robust evidence base, current research suggests that the relatively small number of social firms in the UK offer real jobs that are sustainable and highly valued by employees for providing more mental health-related support than mainstream employment. Further development of social firms may be particularly important given documented implementation barriers to IPS, and low levels of job tenure. The social firm model may offer a viable route to both accessing and staying in employment for people with mental health problems. If they are to become a widely available vocational resource, suitable for the full spectrum of mental health problems including serious mental ill-health, investment and similar levels of state support and legislation as received by social firms in Europe and elsewhere will be required [54, 55].

## Supplementary Information

**Additional file 1.**

## Data Availability

The dataset analysed in the current study are available from the corresponding author on reasonable request.

## Abbreviations

UK: United Kingdom
IPS: Individual placement and support

## Footnotes

1 Data extracts are annotated as follows: E = employee respondent; M = manager respondent; CFG = clinician focus group; I = interviewer.
