# Supplementary material for: Vocational rehabilitation via social firms: a qualitative investigation of the views and experiences of employees with mental health problems, social firm managers and clinicians
Source: BMC Psychiatry. 2021 Nov 12;21:566. doi: 10.1186/s12888-021-03577-5 (PMC8590221; doi:10.1186/s12888-021-03577-5)
Supplement: Supplementary file 1 — Additional file 1. [file 12888_2021_3577_MOESM1_ESM.doc]

**Supplementary Material**

**Vocational rehabilitation via social firms: A qualitative investigation of the views and experiences of employees with mental health problems, social firm managers and clinicians.**

Nicola Morant, Alyssa Milton, Eleanor Gilbert, Sonia Johnson, Nicholas Parsons, Swaran Singh, Steven Marwaha

**Submission ID f3786cd2-0f61-4b57-83c1-112ad27c7314**

Appendix 1: Interview and Focus Groups Topic Guides

**Interview topic guide for social firm employees with mental health problems.**

**Part 1: Setting the scene**

***Use this section to get a general sense of the person’s work and workplace that you can use to orient later questions.***

1. Can you tell me about your current work in this Social Firm?

*Probe-*

- 1. How long have you been working for this company?
  2. What is your job role here?
  3. How many hours a week do you work?

**Part 2: Pathways into social firms**

1. How did you find out about this job?
2. What were some of the reasons you came to work here?
3. Can you tell me about your experience of the recruitment process?
   1. Was there an advert? If so what caught your eye?
   2. Can you tell me about the application process?
   3. Did you come for an interview? *If yes* can you tell me about the interview?
   4. How well did the interviewers allow for your mental health problems/experiences of mental distress?
4. Overall, how did you find this recruitment process?
5. Are there any ways you think the recruitment process could be improved?
   1. If so, can you say what these are?

**Part 3: Experience of working in Social Firm**

1. How do you find working here?
2. How important is this to you that this business is a social firm?
   1. If it is important, why?
3. Has your job changed at all since you started here?

*Probe-* Changes in hours; types of work or responsibilities; changes from voluntary to paid work; promotion

1. How much opportunity in the company is there to:
   1. Change job roles
   2. Go into management

5. How much control do people have over their jobs?

6. How much input do employees have into the company aims?

7. To what extent is mental health openly discussed here?

Probe -

1. Do the other workers know you have a mental health diagnosis?
2. Do you know about the mental health issues of other workers?

8. How are mental health needs supported here?

9. Can you recall any instances when you have needed support at work for your mental health?

*If yes, Probe -*

1. What happened?
2. How was it dealt with or supported within this company?
3. How did you feel about this?

10. How have any of your experiences of discussing or supporting your own or others mental health issues affected your relationships with the people you work with such as:

1. Other workers?
2. Your boss?
3. The people you manage? (if applicable)
4. How do you feel about this?

11. How well does the company support people with a mental health diagnosis/ in mental distress generally?

12. How well do you think the company promotes equal opportunities?

13. Is there anything more that companies like this one could do to support people with a diagnosis of mental health problems with work?

14. Is there anything that you feel this Social Firm could do differently? Perhaps better *Probe –* In general; to support people with a mental health diagnosis/ in mental distress?

**Part 4: Previous work**

1. Can you tell me about any jobs you have had before coming to work for this company? ***Touch gently on this as it can be a sensitive area.*** *If any previous jobs probe: t*ype of work; length of time; full-time / part-time status; was it a company for people with disabilities; can you tell me about your reasons for leaving?

2. Have any of the previous jobs you’ve done been part of a scheme for people with a mental health diagnosis (such as an ‘individual placement scheme’, where individuals are supported to find and keep a job in open employment)? *If so, probe: w*hich programme was it; how did you find the programme? can you tell me a little about the work?

*If any previous jobs*

3. In comparison to working elsewhere, what are any positive aspects of working in a Social Firm like this one?

*If any previous jobs*

4. In comparison to working elsewhere, what are any problems of working in a Social Firm like this one?

1. Would you recommend working in a Social Firm like this one to someone with a diagnosis of mental health problems? *Probe reasons*

6. Would you recommend working in a social Firm like this one to someone who may be disadvantaged in other ways as well? *Probe reasons*

***Instructions to interviewers: Only ask the next question if the person has already mentioned experience of an IPS scheme in the introductory questions.***

7. How does working for this company compare to your experience of IPS?

**Part 5: Impact on life**

1. In what ways has working here affected other areas of your life?

*Probe - q*uality of life; learning new skills; financial situation; views of your future hopes, fears and aspirations; social networks /friendships; mental health symptoms/mental distress; treatment; medication; how you feel about yourself; any stigma you have faced because of your mental health diagnosis.

**Part 6: Plans or views about the future**

1. How do you see your future working here?

2. How would you feel about working outside a social firm?

3. If you did move on from here what type of employment would you like to have?

4. Is there anything more you would like to add?

- **Thank you!**

**Topic Guide for Focus Group and Interviews with Social Firm Managers**

1. What are the benefits for people with mental health problems (MHP) of working in social firms?

Prompt: in comparison to work outside of the sector

1. What are the benefits for the workplace?
2. What are the problems for people with MHP when working in social firms?

Prompt: in comparison to work outside of the sector

1. What are the main challenges in managing staff with MHP? What are the benefits?
2. What process to you have in place to support employees with MHP?

Prompt: do these ever cause challenges?

1. Are social firms suitable for people with mental health problems with a range of severities and disability levels?

Prompt: with different diagnosis, previous employment histories or risk profiles?

1. Are there typical accommodations you make for people? If yes, what are they?
2. Do you have any mental health training?

Probe: How useful is this/ would this be?

1. How do you deal with people becoming unwell and with sickness leave?

Probe: Is this informal or written into policies and procedures?

1. Is there ever tension between supporting disadvantaged workers and running a business? If yes, can you give some examples?
2. Do you or you firm have contact with mental health services?

Prompt: what is this like? How could this be improved?

1. Does your recruitment process allow you to accept all people with MHP into the workplace?
2. Is there anything else you would like to add that has not been discussed?

**Topic Guide for Focus Groups with Mental Health Professionals**

*Group discussion was preceded by a short presentation by group facilitators describing social firms in the UK with some case study examples.*

- Are there any Social Firms locally that employ people with mental health problems?
- If yes, what knowledge do you have of these?

Prompt: either direct contact or information from service users or others

- What is your past experience of these?
- How does the Social Firms model compare to Individual Placement and Support which helps people obtain open market work and then provides ongoing support?
- What might the benefits be for people with mental health problems in working in Social Firms in comparison to other types of employment? (Probe: obtaining employment, stigma, disclosure, recovery)
- What might be the problems of working in Social Firms for people with mental health problems in comparison to other types of employment? (Probe: moving on to work outside the Social Firm sector, recovery
- What are the types of people on your caseloads for whom Social Firms have been or would be most suitable?

Probe: motivation, disability level, diagnoses, risk history, work history. Why?

- How could Social Firms recruit people with mental health problems?
- Does your Trust have links with any Social Firms?
  - How does this work
  - What are the benefits of this
  - What are the difficulties
  - Which mental health professionals are best placed to develop these links
- Do you have any experiences of linking with other types of employment scheme?
  - How does this work
  - What are the benefits of this
  - What are the difficulties
  - Could these type of links be developed with Social firms?

Appendix 2: COREQ 32-item Checklist

Authors: 1. Nicola Morant; 2. Alyssa Milton, 3. Eleanor Gilbert, 4. Sonia Johnson, 5. Nicholas Parsons, 6. Swaran Singh, 7. Steven Marwaha.

| **Number** | **Item** | **Description** | **Page No.** |
| --- | --- | --- | --- |
| 1. | Interviewer | Author 2 and 3 conducted the focus groups and interviews with social firms managers and clinicians. Eight service user researchers conducted interviews with social firm employees with training, support and supervision from authors 1, 2 and 3. These people are acknowledged in the Acknowledgements section | Page 6 and 7 |
| 2. | Researcher credentials | Author 1: PhD  Author 2: BSc, MAppSc, PhD  Author 3: BA  Author 4: BA, MSc, MRCPsych, DM.  Author 5: PhD  Author 6: BA, MBBS, MRCPsych, MA, MSc, PhD  Author 7: MRC Psych, PhD | n/a |
| 3. | Occupation | Author 1 is an associate professor specializing in qualitative research in mental health. Author 2 is a psychologist and research fellow specializing in qualitative and mixed methods research and has experience working with mental health focused social firms. Author 3 is an experienced research assistant. Author 4 and 6 are professors and consultant psychiatrists both with extensive expertise in mixed methods research. Author 5 is an associate professor and statistical advisor. | Title page |
| 4. | Gender | Authors 1, 2, 3 and 4 are female. Authors 5, 6 and 7 are male. | n/a |
| 5. | Experience and training | Authors are experienced and active researchers with expertise in qualitative, quantitative, and mixed methods approaches. Authors have researched and published in the broad topic area previously. | References 3-5, 8, 31, 41 |
| 6. | Relationship established | Employees and managers of social firms in England and Wales employing people with mental health problems that had previously participated in our initial quantitative survey were advised of the current study through their social firm communications. Employees and managers who were interested in participating contacted either author 2 or 3 directly, or the social firm manager.  For clinician focus groups, clinicians were recruited from community mental health teams in the Midlands of England and London that were willing to take part. Recruitment was via managers of these teams, with clinicians informing managers if they were interested in participating. Managers subsequently advised author 2 or 3 directly.  Prior to interviews and focus group, participants had the opportunity to review participant information and consent forms, and discuss any questions, before giving informed consent. Interviews with social firm employees were conducted by service user researchers, in order to enhance rapport and a relationship built on mutual understanding. | Page 6 and 7 |
| 7. | Participants’ knowledge of the interviewer | Interviewers had no previous professional or personal relationship with any of the participants. Co-authors who did not conduct interviews read de-identified transcripts, thus had no personal relationship with, or knowledge of participants. Participants were informed about who the involved researchers were in the Participant Information Statement (PIS). | n/a |
| 8. | Interviewer characteristics | Interviews with social firm employees were conducted by a team of 8 service user researchers. The rational for this was to assist in establishing rapport and a relationship built on mutual understanding. Extensive qualitative training, supervision and support were provided by authors 1, 2 and 3 to support the process.  Focus groups and interviews with social firm managers and mental health clinicians were conducted by a psychologist and researcher with experience in qualitative and mixed-methods research in mental health settings (Author 2) and by a research assistant with extensive experience in mental health interventions (author 3). Author 2 had international experience working with mental health focused social firms and community mental health teams. This prior knowledge helped to enrich the data collection and its interpretation during analysis. | Page 7 |
| 9. | Methodological orientation and theory | Within an epistemological framework of critical realism, we analyzed data using thematic analysis. Our analysis combined inductive and deductive approaches, allowing us to explore predetermined questions such as reasons for reported levels of work satisfaction, as well as to explore concepts and meanings within the data more inductively. | Page 7 |
| 10. | Sampling | We aimed for maximum variation sampling using the following processes: In order to obtain a diverse data sample that reflected the diversity of social firms in the UK and of their employees with mental health problems, we contacted all 33 eligible social firms in England and Wales that had been previously identified in our national survey to request participation in this study. Of these, employees and / or managers from 14 social firms participated, and we obtained data from 23 employees and 12 managers. We collected data from the employee sample on their demographic and clinical characteristics and on the nature of their social firm employment. This allowed us to assess how this sample compared on these characteristics to the larger sample of employees of all social firms in England and Wales we had surveyed in previous work [Gilbert et al, 2013]. This comparison showed that it was similar on demographic and clinical characteristics, although there was lower representation of people with a diagnosis of schizophrenia or psychosis.  Mental health clinicians were sampled from a range of community mental health services, 3 in London and 2 in The Midlands of England. In order to obtain a diverse sample of clinicians from a range of professional backgrounds, recruiting service managers were asked to suggest clinicians from a range of professional backgrounds. We collected data on the types of community mental health services and professional backgrounds of clinician participants. We did not collect data on the demographic or professional characteristics of social firm managers, so were not able to comment on the composition of this sub-sample. This is acknowledged in the study limitations. | Pages 6, 7, 9, 17 and 20 |
| 11. | Method of approach | Mangers of social firms identified in our previous national survey sent communications to their employees advising of the study and could volunteer to participate themselves. Mental health clinicians were made aware of the study via the community mental health services they worked in and were recruited via mental health team managers. | Pages 6 and 7 |
| 12. | Sample size | Sample sizes for the three groups of participants were planned based on published guidance [Guest et al, 2006]. As we wanted to prioritise the experiences of social firm employees and to include the perspectives of people working in a range of employment sectors, and with variable lengths of employment and forms of mental ill-health, we planned to interview up to 30 social firm employees. For social firm managers and clinicians we used guidance of around 12 as a minimum sample size [Guest et al, 2006]. Our final sample sizes were 23 employees with mental health problems, 12 managers of social firms, and 16 mental health clinicians. | Page 8 |
| 13. | Non-participation | No social firm employees, managers or clinicians chose not take part in interviews or focus groups after reading the participant information statement, and no-one withdrew their data subsequently. All interviews that commenced were completed. | n/a |
| 14. | Setting of data collection | Interviews with social firm employees were conducted face-to-face at social firms. A focus group with mangers of social firms was held at the Social Firms UK conference, and additional phone interviews were conducted with social firm managers who could not attend the focus group. Clinician focus groups were conducted at community mental health service and university settings. | Page 7 |
| 15. | Presence of non-participants | Participants could choose to have a support person or advocate present at the interview, however this was not required for any interviews. | n/a |
| 16. | Description of sample | Twenty three employees of 11 social firms in England with mental health problems; twelve managers of social firms in England; sixteen clinicians working in community mental health teams in London and the Midlands. | Page 9 and Tables 1 and 2 |
| 17. | Interview guide | Employees with mental health problems: Interviews were semi-structured. Questions covered perceived benefits and problems, recruitment and support mechanisms, impact on other areas of life, comparisons with previous work experiences, and aspirations for the future.  Social firm managers: The focus group explored the benefits and problems of social firm employment for people with mental health problems and for the social firm, and links with mental health organizations. Individual interviews covered the same topics.  Mental health clinicians: Focus groups explored clinicians’ awareness of local social firms and experiences of supporting service users to work there, how social firms compare to other vocational models, and the suitability of social firms for their service users. | Pages 6-8, and Appendix 1 |
| 18. | Repeat interviews | n/a | n/a |
| 19. | Audio/visual recording | Interviews with social firm employees and managers, and focus groups with mental health clinicians were audio recorded. For the focus group with social firm managers, detailed notes of the discussion were taken and their accuracy was checked with participants. | Page 7 and 8 |
| 20. | Field notes | Detailed notes were made in the social firm managers’ focus group. These were updated during researcher discussions to inform analysis. | Page 7 |
| 21. | Duration | Individual interviews lasted 30-60 minutes. Focus groups lasted between 60 and 75 minutes. | Page 7 |
| 22. | Data saturation | Interviews were continued until the team considered that data saturation had been reached. This was discussed and agreed upon by the team of researchers (author 1, 2 and 3), in consultation with the other authors and the lived experience researchers. | Page 9 |
| 23. | Transcripts returned | Transcripts were not returned to participants. | n/a |
| 24. | Number of data coders | Data was coded by authors 1, 2 and 3. Other members of the research team contributed to discussions about theme development. | Page 8 |
| 25. | Description of the coding tree | Following the broad principles and stages of thematic analysis, data codes, themes, and subthemes were iteratively developed and refined throughout the analytic process. Descriptions of the themes, subthemes and codes were developed and captured in a coding framework in Nvivo software. | Page 8 |
| 26. | Derivation of themes | Our analysis combined inductive and deductive approaches, allowing us to explore predetermined questions such as reasons for reported levels of work satisfaction, as well as to explore concepts and meanings within the data more inductively. | Page 8 |
| 27. | Software | NVivo 10 | Page 8 |
| 28. | Participant checking | Participant checking did not take place. Instead a service user researcher who had also conducted some interviews with social firm employees was involved in team discussions about theme development and overall data analysis. | Page 8 |
| 29. | Quotations presented | Illustrative quotes from participants are included. Quotations are identified with participant type and number to ensure anonymity. Specifically, E=employee of social firm with mental health problem, M= manager and C= clinician. | Pages 9-17 |
| 30. | Data and findings consistent | Data and findings are consistent throughout the manuscript. Social firm employees and managers provided ‘inside’ perspectives on working in social firms and there were many thematic similarities in their data. Accordingly, findings from employees and managers are presented together, followed by analysis of clinicians’ ‘outside’ perspective. | Pages 9-17 |
| 31. | Clarity of major themes | Results are structured according to major themes. The three major thematic sections of the Results are:   - The Supportive Ethos of social firms - Dynamic Issues: Change and progression over time - Clinicians’ Views (an ‘outside’ perspective)   Figure 1 provides an overview of the sub-themes within the first two major themes, and how these are related. | Pages 9-17 |
| 32. | Clarity of minor themes | Subthemes are presented for two of the major themes (1. The supportive ethos of social firms; and, 2. Dynamic Issues: Change and progression over). Sub-themes are organized and detailed in sub-sections of the Results and named in sub-heading. Within each sub-section, employee and manager positions are compared and where minority positions were detected these are noted. Figure 1 also summarizes how themes and sub-themes are inter-related. The sub-themes described in the Results are as follows:   - The supportive ethos of social firms theme encompassed three sub-themes: openness about mental health issues; flexibility; management, team and peer support. - Dynamic Issues: Change and progression over time theme encompassed three sub-themes: Social firms facilitate return to work; Wider Impacts; Social firms as stepping stones or destinations? | Pages 9-17 |
